# Bacterial RNA polymerase caps RNA with various cofactors and cell wall precursors

**DOI:** 10.1093/nar/gkx452

**Published:** 2017-05-22

**Authors:** Christina Julius, Yulia Yuzenkova

**Affiliations:** Centre for Bacterial Cell Biology, Institute for Cell and Molecular Biosciences, Newcastle University, Baddiley-Clark Building, Richardson Road, Newcastle upon Tyne, NE2 4AX, UK

## Abstract

Bacterial RNA polymerase is able to initiate transcription with adenosine-containing cofactor NAD+, which was proposed to result in a portion of cellular RNAs being ‘capped’ at the 5′ end with NAD+, reminiscent of eukaryotic cap. Here we show that, apart from NAD+, another adenosine-containing cofactor FAD and highly abundant uridine-containing cell wall precursors, UDP-Glucose and UDP-N-acetylglucosamine are efficiently used to initiate transcription *in vitro*. We show that the affinity to NAD+ and UDP-containing factors during initiation is much lower than their cellular concentrations, and that initiation with them stimulates promoter escape. Efficiency of initiation with NAD+, but not with UDP-containing factors, is affected by amino acids of the Rifampicin-binding pocket, suggesting altered RNA capping in Rifampicin-resistant strains. However, relative affinity to NAD+ does not depend on the −1 base of the template strand, as was suggested earlier. We show that incorporation of mature cell wall precursor, UDP-MurNAc-pentapeptide, is inhibited by region 3.2 of σ subunit, possibly preventing targeting of RNA to the membrane. Overall, our *in vitro* results propose a wide repertoire of potential bacterial RNA capping molecules, and provide mechanistic insights into their incorporation.

## INTRODUCTION

For few decades multi-subunit RNA polymerase (RNAP) from *Escherichia coli* was known to be able to start RNA synthesis with cellular nucleotide coenzymes, adenosine derivatives NAD+ (nicotinamide adenine dinucleotide), NADH (reduced form of NAD+) and FAD (flavin adenine dinucleotide) (1). Authors of this work suggested that cofactor moiety could function analogously to the eukaryotic mRNA cap. Until very recently, the physiological significance of this discovery remained obscure, and the accepted view was that non-processed bacterial RNAs carry 5′ triphosphate. In the last few years however, the data started to accumulate that some cellular RNAs carry cap-like structure—*E. coli* and *Streptomyces venezuelae* bear NAD+ at the 5′ end (2). In 2015 those RNAs in *E. coli* were captured via 5′ NAD+ moiety and identified by next generation sequencing (3). It transpired that those RNAs were mainly regulatory sRNA and some mRNAs. Only relatively small proportion of the whole population of the particular RNA was NADylated *in vivo*. The most heavily NADylated were RNAI and CopA, abundant short antisense RNAs controlling pUC19 plasmid replication. Notably, for the NADylated transcripts with known start site, the +1 position coded for A, suggesting that it is RNAP incorporates NAD+ at the 5′ end of RNA via mechanism shown earlier, rather than some post-transcriptional mechanism being involved (2). While our paper was in preparation, biochemical and structural data using specific promoter assays has been published that confirmed the promoter-dependent and sequence-specific incorporation of NAD+, NADH and 3′- dephosphocoenzyme A (dpCoA) (4). Correlation between efficiencies of NAD+ incorporation *in vitro* and the extent of NADylation *in vivo* on two different promoters suggested that transcription might be the main, if not the only, capping mechanism. Crystal structures of initiation complex containing dinucleotide RNA products (to avoid confusion, here and after, we refer to the RNA length counting NAD+ and other dinucleotide co-factors as a single nucleotide) initiated with adenosine triphosphate (ATP), NAD+ and dpCoA were solved for *Thermus thermophilus* RNAP (4) demonstrated that, apart from interactions common for all three cofactors, contacts of NAD+ moiety additionally include side chains of β subunits residues D516 and H1237. The authors also proposed that nicotinamide moiety of NAD+ may rotate to interact with the −1 position of the template, thus explaining different efficiencies of NAD+ incorporation on different promoters.

Eukaryotic mRNA turnover depends on the efficiency of cap removal. Major catalytic role in decapping in eukaryotes is played by NUDIX motif-containing protein Dcp2p. In bacteria, NudC (NADH pyrophosphohydrolase), which contains NUDIX motif was shown to de-cap RNAs from NAD+ (5). Notably, NudC has much higher affinity towards NADylated RNA compared to NAD+ itself (5). Existence of decapping mechanism makes the analogy between prokaryotic and eukaryotic RNA processing even stronger.

In eukaryotes mRNA capping plays vital role in RNA degradation, splicing, translation initiation and nuclear export. Physiological significance of bacterial RNA capping is not yet clear. The only role for capping that was put forward and got some experimental backing, is the protection of the transcript from degradation. The data on capped RNA stability are, however, conflicting. Bird *et al.* reported 3- to 4-fold increase of in NADylated RNAI stability in ΔNudC cells (lacking de-capping activity) (4), in contrast to another study, where deletion of NudC did not affect the overall stability of RNAI and GcvB, two RNAs, most heavily NADylated *in vivo* (3). Moreover, some RNAs with high NAD+ cap content are inherently very stable, for example sroB with half-life of more than 32 minutes (6). All these data suggest possible additional roles for prokaryotic capping apart from RNA stability.

To date only ADP analogues were identified as caps, NAD+ and/or NADH. Cells use a number of nucleotide cofactors and these might be just the first identified ones among many substrates used by RNAP for RNA capping. There are several poorly characterized NUDIX hydrolases in *E. coli*, which can potentially serve to remove capping molecules different from NAD+ (7), which have not been found yet.

Here we provide further insights into the mechanism of capping with adenine containing cofactors (NAD+, NADH, NADP+, FAD) and its possible role in transcription. We also show that the repertoire of potential capping molecules is wider, and includes uridine containing precursors of cell wall synthesis (UDP-glucose and UDP-N-acetylglucosamine). Our data also suggest the role of region 3.2 of initiation factor sigma in guarding transcription against incorporation of ‘long-tailed’ NTP analogues into RNA.

## MATERIALS AND METHODS

### Materials

ATP and UTP were from GE Healthcare; AMP, ADP, NAD+, NADH, NADP+, FAD, UMP, UDP, UDG-Glc and UDP-GlcNAc were from Sigma Aldrich; UDP-MurNAc pentapeptide was a kind gift from Prof. Vollmer.

### Proteins

Mutations in *E. coli* rpoB gene were constructed by site-directed mutagenesis in polycistronic expression plasmid pGEMABC, coding for RNAP core subunits α, β and β’ (8). Those subunits were overexpressed in *E. coli* T7 express strain (New England Biolabs) together with ω subunits from expression plasmid pRSFD_2_rpoZ (8). Wild-type and mutant RNAPs core enzymes were purified as described in (9). RpoS gene encoding σ^S^ was cloned into expression vector pET28, as previously were σ^70^ and σ^70Δ3.2^ (10). N-terminal Hisx6-tagged σ^70^, σ^70Δ3.2^, σ^S^ were expressed from overexpression vector pET28 and purified as described in (11).

### *In vitro* transcription

A total of 0.3 pmols of wild-type or mutant RNAP core with 1 pmols of σ^70^ (wild-type or mutant) or σ^S^ and 2 pmols of promoter-containing linear DNA fragment were incubated at 37°C for 10 min in 10 μl of transcription buffer (20 mM Tris–HCl (pH 7.9), 40 mM KCl, 0.1mM ethylenediaminetetraacetic acid) at 37°C, then nucleotides or nucleotides analogues were added to the final concentration of 500 μM (unless otherwise indicated). Transcription was initiated by the addition of 10 mM MgCl_2_, 50 μM (α^32^P)-CTP, 12.5Ci/mmol (Hartmann Analytic) for RNAI and acnA templates; for T7A1 template 50 μM (α^32^P)-UTP, 12.5 Ci/mmol of were used. Reactions were stopped after 3 min incubation at 37°C (unless otherwise indicated) by the addition of formamide-containing loading buffer. For the kinetics of synthesis of 9 nt-long transcript from RNAImod, 500 μM ATP or NAD+ were incubated with promoter complex and then 20 μM UTP and 20 μM (α^32^P)-CTP, 12.5 Ci/mmol were added in the absence or presence of 5 μg/ml of Rifampicin. Reactions were stopped after periods of time indicated on Figure 4A. Products were separated on denaturing polyacrylamide gels (30% acrylamide, 3% bis-acrylamide, 6M urea, 0.5 Tris-borate EDTA buffer), revealed by PhosphorImaging (GE Healthcare), and analysed using ImageQuant software (GE Healthcare). For 9-nt long RNA synthesis in Figure 2D on RNAImod template 500 μM of either ATP, NAD+, NADH, NADP or FAD were incubated with promoter complex and then 25 μM UTP and 25 μM (α^32^P)-CTP, 12.5 Ci/mmol, were added and reactions were incubated for 10 min. For similar experiment on Figure 5C to synthetize 12-nt long RNA on acnA template 500 μM of either UTP, UDP-Glc or UDP-GlcNAc were incubated with promoter complex and then 25 μM ATP and 25 μM (α^32^P)-CTP, 12.5 Ci/mmol, were added and reactions were incubated for 10 min. For apparent *K*_m_ determination on RNAImod and acnA templates NTPs and analogues were used in concentrations ranging from 10 μM to 10 mM and constant 50 μM CTP (second NTP for both templates) concentration. Reactions were incubated for periods of time chosen to get approximately the same intensities of the product bands. The bands intensities were quantified using ImageQuant software; to calculate the initial reaction rate these numbers were divided by reaction duration time. These data were fitted to hyperbolic equation using non-linear regression procedure in SigmaPlot software.

## RESULTS

### *Escherichia coli* RNAP is able to initiate transcription using adenosine diphosphate analogues

**Figure 1.**
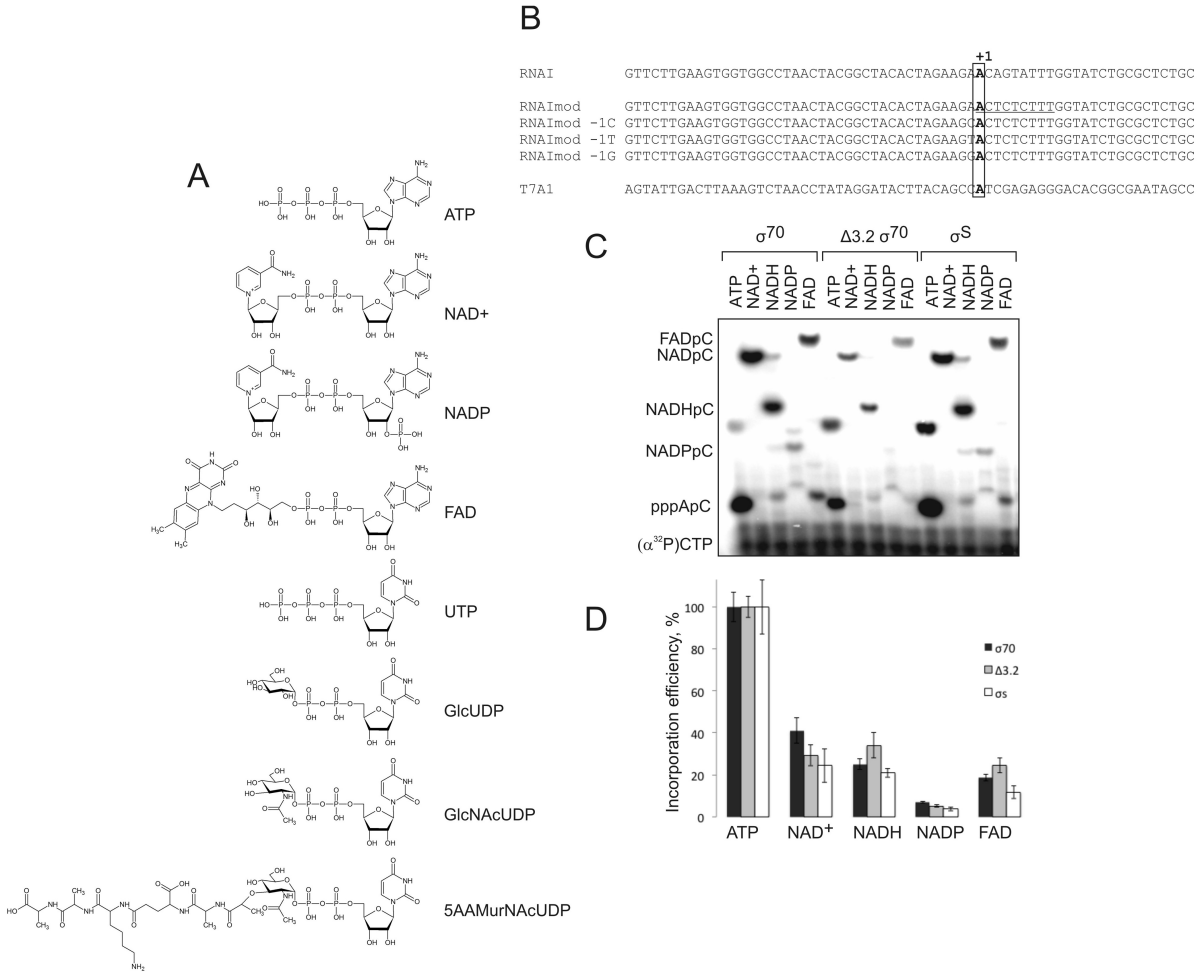
ADP-related cellular cofactors are utilized by RNAP for initiation of transcription. (**A**) Structures of ATP, NAD^+^, NADP^+^, FAD, UDP, UDP-Glc (GlcUDP), UDP-GlcNAc (GlcNAcUDP) and UDP-MurNAc pentapeptide (5AAMurNAcUDP). (**B**) Templates (partial sequence of non-template strand around transcription start site) used for *in vitro* experiments containing T7A1, RNAI promoters and RNAI template with modified initially transcribed sequence, RNAImod. +1 position for all templates is in bold, −1 position is framed, 9 nt initially transcribed sequence in RNAImod is underlined. (**C**) Initial transcript synthesis on RNAImod template using RNAP holo ^σ70^, holo ^σS^ and holo^σ70Δ3.2^ and 500μM ATP, NAD^+^, NADH, NADP^+^ and FAD as initiating substrates and (α^32^P)-CTP as the next nucleotide. (**D**) Plot reflecting incorporation efficiencies for alternative substrates in percentage from efficiency of ATP incorporation for RNAP holo ^σ70^, holo ^σS^ and holo^σ70Δ3.2^ on RNAImod, the values are an average of the three independent experiments, error bars represent plus and minus one standard deviation.

**Figure 2.**
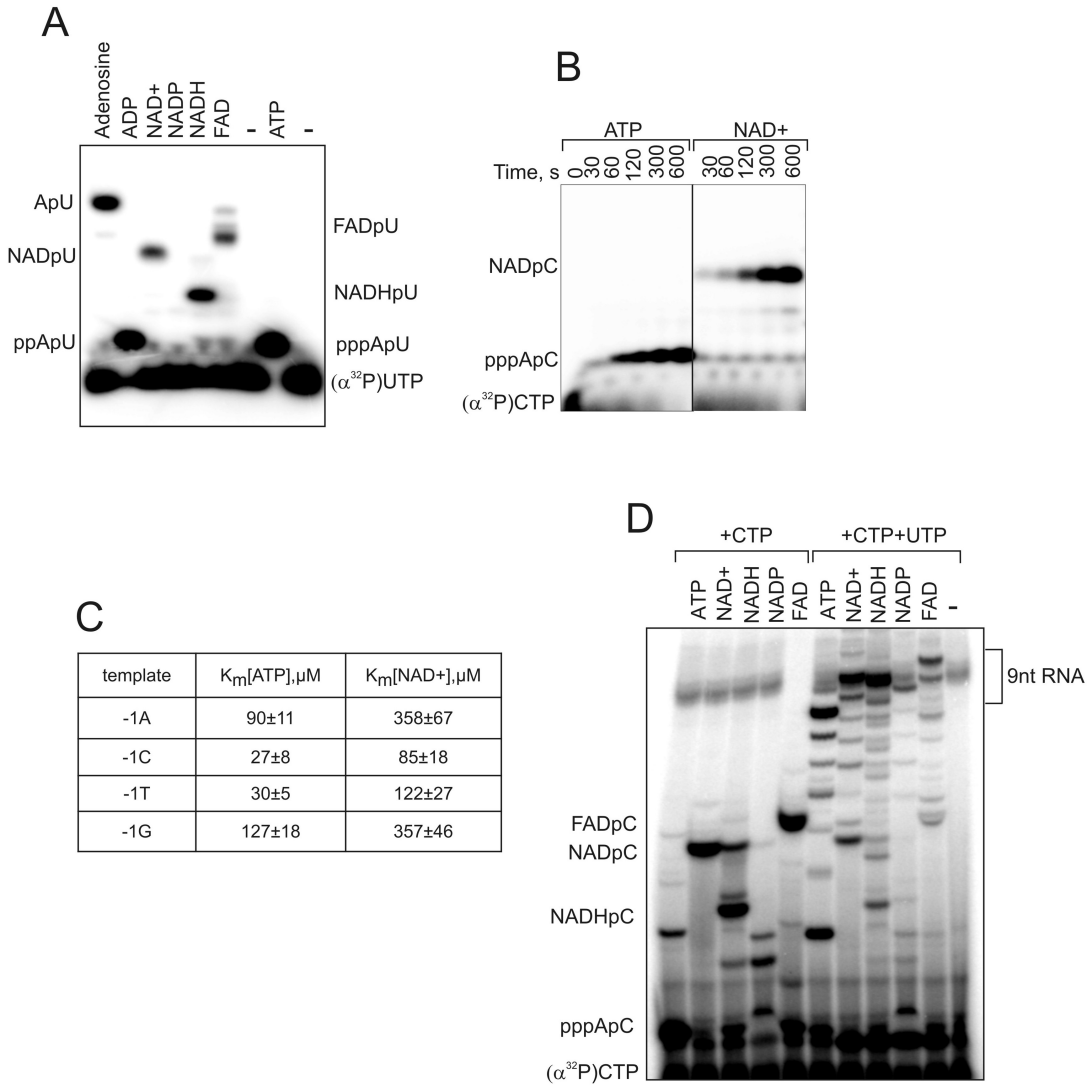
Biochemical characterization of the ADP analogues incorporation into a transcript. (**A**) Initial products synthetized using ATP and cofactors as initiating substrates on T7A1 promoter template with RNAP holo^σ70^*Escherichia coli* and (α^32^P)-UTP as next nucleotide. (**B**) Kinetics of initial product synthesis on RNAImod template with RNAP holo^σ70^ with either ATP or NAD+ as initiating substrate. (**C**) *K*_m_ for ATP and NAD+ as initiating substrates measured on RNAImod templates with different identity of −1 template bases (sequences in Figure 1B); numbers that follow the ±sign are errors that are standard deviations of the fitting. (**D**) Transcripts initiated with ATP, NAD^+^, NADH, NADP^+^ and FAD, are elongated to 9 nt transcript on RNAImod template.

Abortive production of short oligonucleotides is the very first stage of the synthesis of the RNA. These products can be resolved in high density denaturing polyacrylamide gel. We analysed the efficiency of incorporation of potential cap molecules into RNA in abortive synthesis by *E. coli* RNAP σ^70^ holoenzyme on a linear DNA containing promoter RNAI (that was shown to be the most heavily modified by NAD+ in *E. coli*). ATP or co-enzymes (each at 500 μM concentration) (structures on Figure 1A) were extended by (α^32^P)-CMP (at 50 μM). Position +3 of the RNAI promoter was mutated to T to make RNAImod template (here and after we refer to the sequence of the non-template strand) to preclude extension of RNA further than position +2, which would obscure kinetics analysis (Figure 1B). As can be seen from Figure 1C, NAD+, NADH, FAD can be efficiently incorporated into the 2-nt long transcript. The results are consistent with the recently published work (4) and the earlier study (1). Efficient incorporation of these coenzymes into the transcript is not a particular property of RNAI, as they were similarly efficiently extended by (α^32^P)-UMP on a strong promoter A1 from bacteriophage T7, where transcription also starts with A (Figure 2A). NADP+ was incorporated very inefficiently into the transcript on both templates, likely due to the 2′-phosphate group sterical hindrance during phosphodiester bond formation. At 1 mM initiating NAD+ and ATP on RNAImod template, the kinetics of formation of NAD+pC was comparable (∼2 times slower) to that of pppApC (Figure 2B). The*K*_m_s for ATP, NAD+ and NADH in initiation on RNAImod template were 90 ± 11 μM, 358 ± 67 μM and 380 ± 72 μM, respectively, which is much lower than their cellular concentrations (see ‘Discussion’ section).

In the presence of CTP and UTP, the short transcripts initiated with NAD+, NADH and FAD on the RNAImod template were efficiently extended into 9-nt products (Figure 2D). The amounts of 9-nt products were comparable to those initiated with ATP, suggesting that, similar to NAD+ capping, NADH and FAD capping may exist in the cell. Note also that the efficiency of abortive products extension (the ratio between 9-nt RNA and dinucleotides) in the case of co-factors was higher than in the case of ATP, suggesting that co-factors increase efficiency of promoter escape (see below).

### The −1 position of template does not influence the relative efficiency of NAD+ versus ATP incorporation into the transcript

Nicotinamide moiety may, in theory, form base pair with template DNA base at the position −1. This may change specificity of NADylation at promoters with particular base at −1 position. It was observed that A to C (non-template strand) change in −1 position decreased the overall efficiency of NADylated transcript synthesis (4). To test if the base at position −1 affects the affinity to NAD+, we used linear templates with RNAImod promoter variants with changes in the −1 position (Figure 1B). We measured apparent *K*_m_ for NAD+ and ATP on these four promoters variants in abortive synthesis as above (Figure 2C). We found that*K*_m_ for NAD+ incorporation in transcript was lower for templates with C and T in −1 position compared with values for −1 A or G. However, the same tendency was observed for incorporation of ATP, suggesting that position −1 of template does not specifically affect affinity to NAD+. This result is consistent with crystal structures of the initiating complexes where initiating NAD+ makes the same contacts with template DNA as initiating ATP (4). Therefore it is likely that different steps of initiation, rather than direct interaction with the template, may influence the relative efficiency of initiation with NAD+ on templates with different −1 position (4). Note that the measured *K*_m_s for NAD+ on either of the templates are much lower than the cellular concentrations (see ‘Discussion’ section).

### Core RNAP determinants for NAD+ incorporation

**Figure 3.**
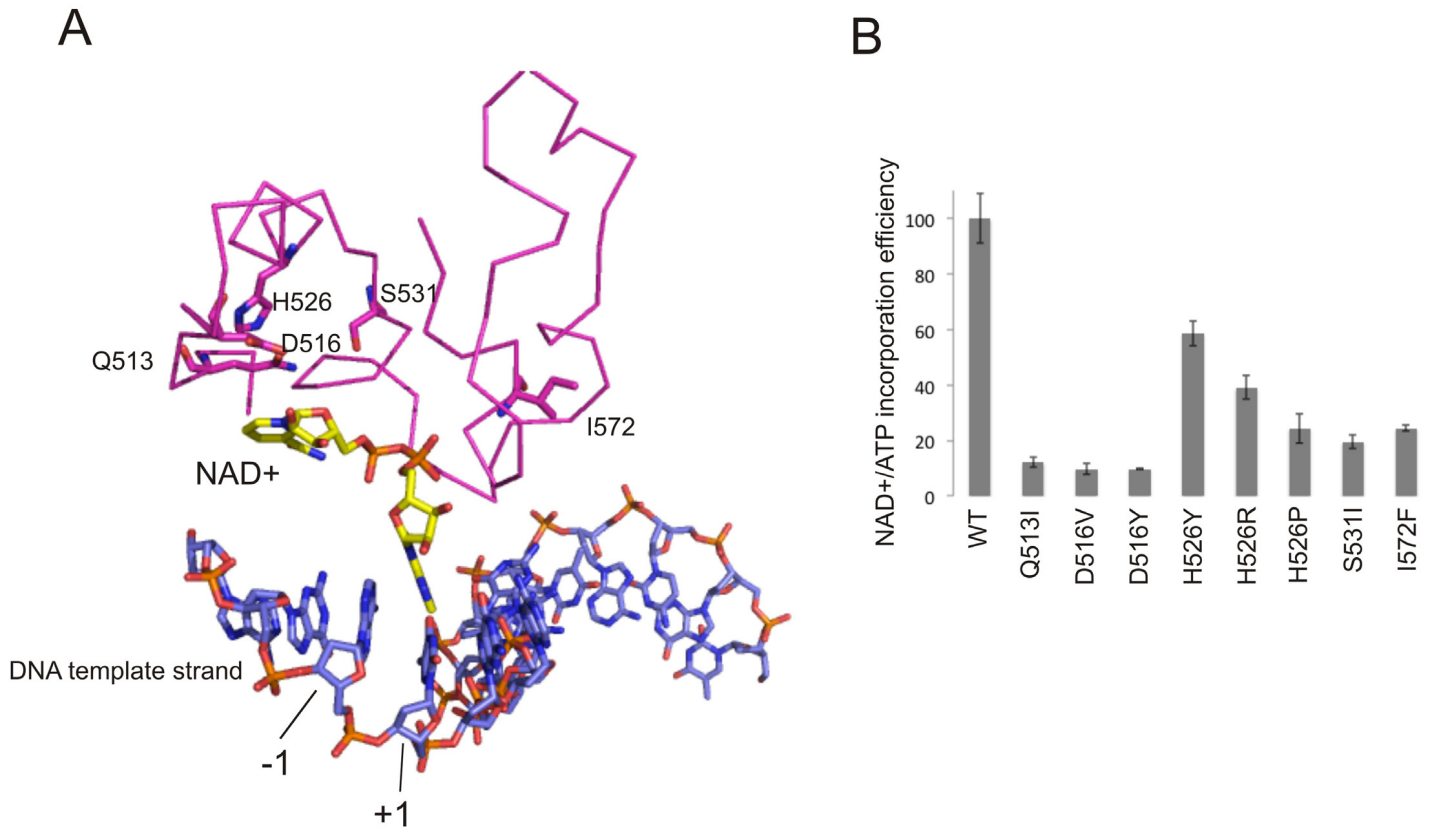
Aminoacids of Rifampicin-binding pocket of β subunit of RNAP influence incorporation of ADP analogues into a transcript. (**A**) Crystal structure of the initiation complex with NAD^+^pC product with *Thermus thermophilus* RNAP, adapted from PDB ID: 5D4D (4). The amino acids of Rif-pocket, whose changes were tested *in vitro* are in purple (*Escherichia coli* numbering), NAD^+^ is in yellow, DNA is in blue, −1 and +1 positions of the templates are indicated. (**B**) Relative efficiency of incorporation of 500 μM NAD^+^ versus ATP into dinucleotide product on RNAImod template by WT and mutant RNAPs with aminoacid changes in β subunit indicated below the plot. The values are an average of the three independent experiments, error bars represent plus and minus one standard deviation.

We tested if there are any other structural determinants for cofactors incorporation apart from −1 position of template. 5′ NTP of the growing transcript passes through the Rifampicin-binding pocket of the β subunit of RNAP. It was shown that γ-phosphate of the initiating ATP is in proximity to the β subunit fragment between amino acids 516 and 540 (12), suggesting where the nicotinamide group of NAD+ could be bound during initiation. We tested several RNAPs with amino acid changes in Rifampicin-binding pocket (Rif pocket) — Q513L, F514A, D516V, D516Y, H526Y, H526R, H526P, S531L, N568A, I572F on RNAImod promoter with either NAD+ or ATP as an initiating substrate. The activities of the enzymes in abortive synthesis varied significantly (either due to specific activities of the enzymes or distortion of the Rif pocket). Figure 3B shows relative efficiencies of NAD+ incorporation (NAD+/ATP ratio) by the mutant enzymes in percentage from that of the WT RNAP. Indeed, in agreement with our hypothesis that part of NAD+ maybe bound in the Rif pocket, the effect was dependent both on the position and nature of the amino acid substitution. The strongest effect on NAD+ utilization was produced by mutations of D516 of β (Figure 3B). This corroborates the crystal structure of the initiating complex with NAD+ primed RNA product, where the D516 side chain is in close proximity of the nicotinamide moiety of the NAD+ (4) (Figure 3A). Changes of other aminoacids that are too far to interact with NAD+ directly, perhaps affect the overall shape of the Rif-pocket.

### Initiation factor does not play a role in selectivity of ADP-containing co-enzymes as substrates

A larger proportion of RNAI was found to be NADylated in stationary growth phase compared to the exponential phase (3). In stationary phase most transcripts are made by RNAP holoenzyme containing σ^S^, while housekeeping σ^70^ operates in exponential phase. We analysed if different initiation factors may dictate specificity towards capping cofactors. To answer this question we tested initiation with NAD+ on RNAI promoter with holo σ^70^ and holo σ^S^ (Figure 1C and D). The rates of abortive products formation with ATP, NAD+, NADH, NADP+ and FAD were similar for both RNAPs (Figure 1C and D). *K*_m_ values for NAD+ were also close for both holoenzymes (358 ± 67 μM for holo σ^70^ and 352 ± 88 μM for holo σ^S^) The results suggest that at least the two initiation factors tested do not provide significant specificity towards utilization of NAD+ as initiation substrate.

Previous functional and structural analyses suggested that region 3.2 of σ subunit protrudes towards catalytic site of the RNAP and may contact the 5′ end of short transcripts (10,11,13,14). We tested initiation with NAD+, NADH, NADP+ and FAD (500 μM) on RNAI promoter by holoenzymes containing either wild-type σ^70^ or mutant σ^70^ lacking region 3.2, σ^70Δ3.2^ (Figure 1C and D). We did not observe any significant differences in specificity, suggesting that region 3.2 does not make contacts critical for cofactors’ binding.

### NADylation of transcript stimulates escape of the RNAP from promoter

**Figure 4.**
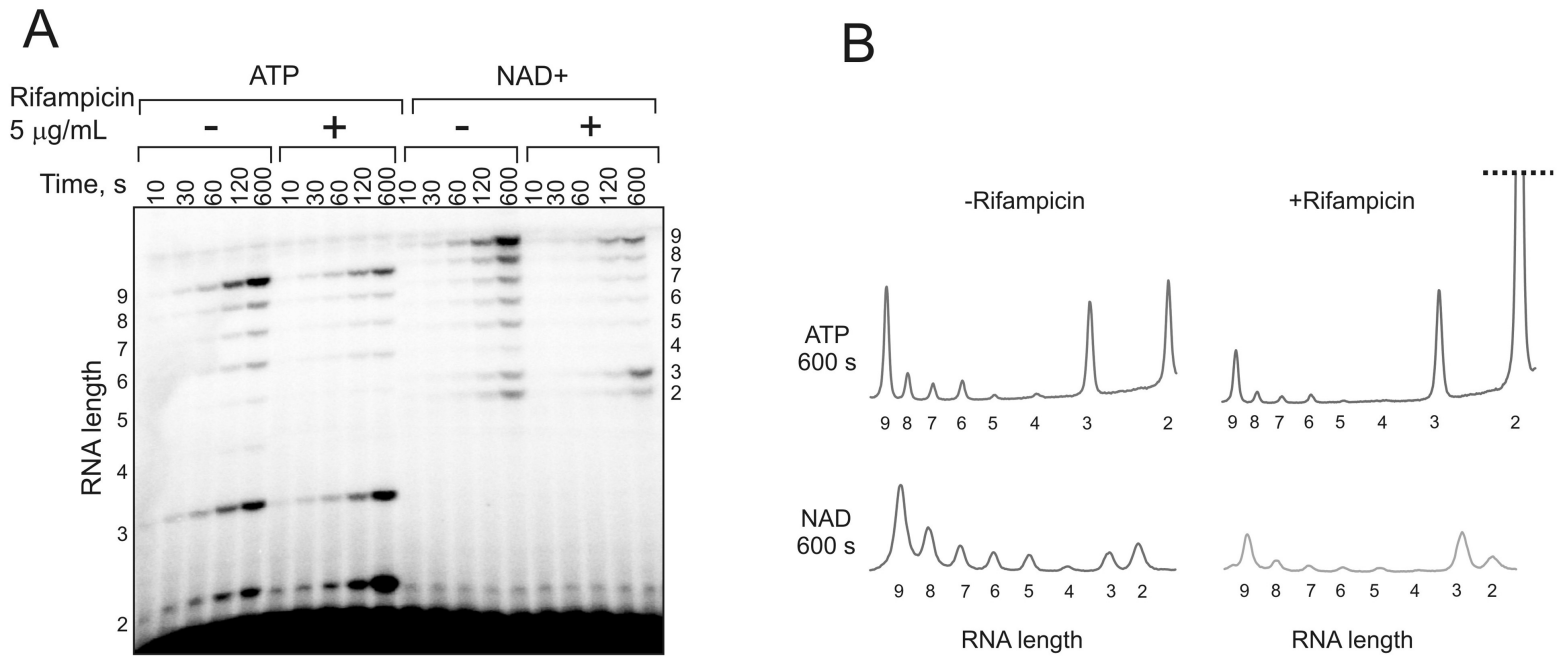
NAD^+^ as initiating substrate improves escape of RNAP from promoter compared to ATP. (**A**) Kinetics of synthesis of 9 nt transcript on RNAImod promoter template (Figure 1B) using either ATP or NAD^+^ at 1 mM, 20 μM (α^32^P)-CTP and 20 μM UTP in the absence or presence of 5 μg/ml of Rifampicin. (**B**) Signal traces taken across the 600 s bands.

From our results it follows that NAD+ interacts differently with RNAP as compared to ATP. These differences might affect stability of the short abortive transcripts, and as a result, their extension during promoter escape. We therefore tested kinetics of 9-nt RNA production initiated with either ATP or NAD+ (500 μM) on RNAImod template. A relatively low concentration of CTP and UTP (20 μM) allowed us to monitor the accumulation of short RNAs ranging from 2 to 9 nt in length. As can be seen from Figure 4A and B there were much less accumulation of the 2- and 3-nt long transcripts when transcription was initiated with NAD+ as compared to ATP (compare the traces of 600 s products in Figure 3B). Rifampicin is known to block escape into elongation, with concomitant increase of abortive synthesis (15). We hypothesized that, if NAD+ containing initial transcripts are bound tighter by RNAP than ATP containing ones, Rifampicin may have different effect on their release. We used low concentration of Rifampicin (5 μg/ml), which was not enough to block transcription completely. Addition of Rifampicin to reaction inhibited production of 9-nt RNA initiated by either ATP or NAD+. Also, as expected Rifampicin increased release of pppApC dinucleotide. However, Rifampicin almost didn’t affect production of NAD+ containing short RNAs, highlighting the difference between promoter escape with ATP and NAD+ (compare the traces of 600 s products in Figure 3B). These results suggest that NAD+ moiety at the 5′end indeed can stabilize short transcripts in the RNAP active centre.

### UDP derivatives can serve as initiating substrates for transcription

Exponentially growing in rich medium *E. coli* cells contain high concentrations of a number of nucleotide analogues potentially capable of initiating transcription. One of the most abundant small molecules in *E. coli* cell, second only to ATP, is UDP-N-acetylglucosamine (UDP-GlcNAc) (16), which participates in formation of peptidoglycan, lipopolysaccharides and teichoic acid cell wall components. We tested if UDP-GlcNAc, along with another precursors of cell wall synthesis, Uridine 5′-diphosphoglucose (UDP-Glc) (structures in Figure 1A) can initiate transcription. We also tested a more complex compound, UDP-MurNAc pentapeptide (Figure 1A), the last-step precursor before the formation of Lipid I, a building block of the cell wall.

**Figure 5.**
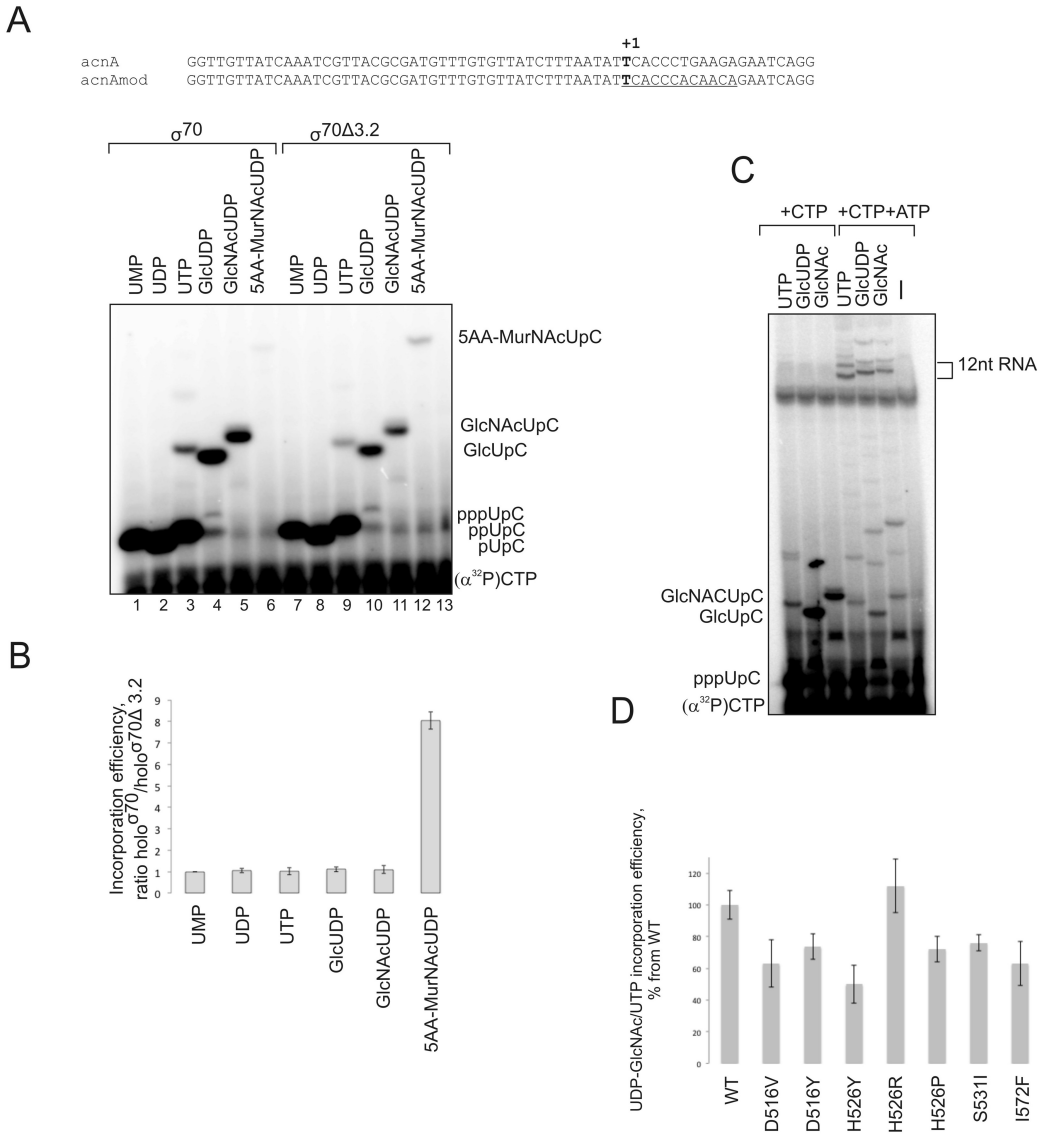
Analogues of UDP can serve as initiating substrates for RNAP *in vitro*. (**A**) Template with acnA promoter (partial sequence around transcription start site) and acnAmod template, +1 position is in bold, 12 nt initially transcribed sequence in RNAImod is underlined. Dinucleotide synthesis on acnA template using either RNAP holo ^σ70^ or holo ^σ70Δ3.2^, and 500 μM UMP, UDP, UTP, UDP-Glc, UDP-GlcNAc and UDP-MurNAc pentapeptide as initiating substrates and (α^32^P)-CTP as the next nucleotide. (**B**) Plot below the gel represents the ratio of incorporation of indicated substrates by holo ^σ70Δ3.2^ versus holo ^σ70^, the values are an average of the two independent experiments, error bars represent plus and minus one standard deviation. (**C**) Transcripts initiated with UTP, UDP-Glc, UDP-GlcNAc, are elongated to 12 nt transcript on acnAmod template. (**D**) Relative efficiency of incorporation of 500 μM UDP-GlcNAc in comparison to UTP into dinucleotide product on RNAImod template by WT and mutant RNAPs with aminoacid changes in Rifampicin-binding pocket of β subunit indicated below the plot. The values are an average of the two independent experiments, error bars represent plus and minus one standard deviation.

In this experiment we used linear templates containing acnA promoter, on which transcription starts with UTP (Figure 5A). We analyzed initiation from UDP-Glc, UDP-GlcNAc, UDP-MurNAc pentapeptide (5AA-MurNAc), along with UMP, UDP and UTP as controls (each at 500 μM concentrations) (Figure 5A) by monitoring synthesis of the dinucleotide transcript after addition of an (α^32^P)-CMP (at 50 μM concentration). As can be seen from Figure 5A, *E. coli* RNAP can efficiently incorporate UDP-Glc and UDP-GlcNAc (comparably to UTP; lanes 3–5). The*K*_m_ values on the acnA promoter for UDP-Glc and UDP-GlcNAc were 300 ± 62 μM and 333 ± 41 μM, respectively (compared to 120 ± 17 μM for UTP). These *K*_m_ values are well below intracellular concentrations of UDP-Glc and UDP-GlcNAc in exponential growth phase (2.5 and 9.2 mM, respectively). We analysed if dinucleotides initiated with UDP-Glc and UDP-GlcNAc can be extended to facilitate escape into elongation. We mutated acnA promoter (acnAmod; Figure 5A) so that we could monitor extension to 12-nt RNA in the presence of the ATP and CTP. As can be seen from Figure 5C, the transcripts initiated with UDP-Glc and UDP-GlcNAc were elongated by RNAP to the 12 nt. Note also, that the efficiency of promoter escape with UDP-Glc and UDP-GlcNAc was higher than that with UTP. These findings suggest that the cell wall precursors UDP-Glc and UDP-GlcNAc may serve as an RNA caps *in vivo*.

Interestingly, UDP-MurNAc pentapeptide was not utilized by RNAP at all. It is possible that RNAP possesses a mechanism that prevents incorporation of UDP-MurNAc pentapeptide into an RNA, which would be too costly for cells and may lead to unwanted targeting of the modified transcript towards the membranes. One of the possible obstacles for the pentapeptide in the active site could be region 3.2 of σ^70^. We therefore analysed incorporation of UMP, UDP, UTP, UDP-Glc, UDP-Glc NAc and UDP-MurNAc pentapeptide (500 μM) by holoenzymes formed with wild-type σ^70^ or σ^70Δ3.2^. As can be seen from Figure 5A, the mutant RNAP indeed acquired partial ability to incorporate UDP-MurNAc pentapeptide, while usage of smaller cell wall precursors was not affected (see also relative efficiencies of dinucleotide formation in Figure 5B). This result suggests that region 3.2 may participate in guarding against incorporation of the cellular nucleotide analogues with long side chains.

To test if Rif-pocket plays role in selectivity of UDP-GlcNAc utilization in initiation, as it does for ADP-containing co-factors, we tested the activities of Rifampicin resistant mutant RNAPs that we used above. Mutant RNAP with Q513I substitution was inactive on acnA even with UTP, and was excluded from analysis. The rest of mutations in Rif-pocket did not affect incorporation of UDP-GlcNAc into the transcript strongly (Figure 5D), suggesting that UDP-GlcNAc may not make specific contacts with amino acids of the Rif-pocket.

## DISCUSSION

Here we showed that RNAP is able to incorporate variety of cellular cofactors at the 5′end of the transcript, suggesting a possibility of a wide repertoire of RNA caps in bacteria. In addition to NAD+ that had been shown to cap some of bacterial RNAs *in vivo*, another adenine containing co-factor FAD can also be incorporated by RNAP in sequence-dependent manner, and thus cap RNAs on +1A (T in the +1 position of the template strand) promoters. Furthermore, we show that UDP-containing cell wall precursors may efficiently cap RNAs on +1U promoters.

The crystal structure of promoter complex with NADpC dinucleotide transcript showed the interactions of nicotinamide moiety with the Rif-pocket of β subunit (4). Indeed, all amino acid substitutions in the Rif-pocket we tested in our experiments specifically decreased the ability of RNAP to incorporate NAD+, thus confirming the structural observations. We observed that substitutions at position 516 are the most detrimental for NAD+ incorporation, consistent with the contacts of nicotinamide moiety with βD516 seen in the structure. Interestingly, Rifampicin resistant substitutions at positions 516, 526 and 531 that strongly inhibited NAD+ incorporation, are those most frequent found in clinic (17). The control of the RNA capping by the Rif-pocket may be involved also after the cap binding in the active centre, at the stage of RNA extension and promoter escape. Indeed, we found that transcription started with NAD+ produced far less of short abortive products than transcription started with ATP on the same promoter.

The crystal structure of the promoter complex with the NADpC revealed that nicotinamide moiety does not interact with the template (4). The authors however observed that a change in the −1 position of the template may change the proportion of NADylated RNAs when both ATP and NAD+ are used in the reaction. They suggested that nicotinamide moiety might change conformation to interact with the template base in −1 position, which has not been captured in the crystals. Although we found that *K*_m_ for NAD+ for promoters with −1C or −1T were lower compared to the promoters with −1G or A, the same tendency was observed for ATP, suggesting that the preference of NAD+ for −1 position is not explained by specific base-pairing of nicotinamide moiety with the −1 position of the template. We therefore suggest that −1 position of the promoter affects the properties of other steps(s) of initiation, thus affecting the efficiency of RNA NADylation.

Region 3.2 of the σ subunit is in close proximity to the Rif-pocket (8) and may serve as another determinant for an efficiency of incorporation of the cofactors. However, we did not find any significant effect of the deletion of region 3.2 on incorporation of either NAD+, NADH, FAD or NADP, as compared to ATP. We also observed no difference in efficiency of usage of these factors by RNAP equipped with either housekeeping σ^70^ versus stationary phase σ^S^ initiation factor. These results suggest that σ subunits do not directly interact with the initiating co-enzymes. We however cannot exclude that other alternative initiation factors can contribute towards or against incorporation of non-canonical capping nucleotides analogues. Furthermore, σ region 3.2 may affect the relative efficiency of capping by its interactions with the template DNA upstream of the start site, thus affecting escape into productive elongation.

We further extended the potential bacterial RNA cap repertoire to derivatives of UDP, the highly abundant precursors of cell wall components. Interestingly we found that while σ region 3.2 did not have effect on the incorporation of UDG-Glc and UDP-GlcNAc, it did inhibit incorporation of a larger UDP-MurNAc pentapeptide. The protection of more complex cell wall precursor from incorporation into RNA may have biological significance. The more complex precursors are more expensive for the cell than initial precursors UDG-Glc and UDP-GlcNAc. Furthermore, modification of the 5′ end of RNA with UDP-MurNAc pentapeptide may potentially lead to targeting of an RNA to a membrane, thus affecting its expression.

*K*_m_ values for NAD+ are in the range of 100–400 μM on various promoter variants, which is below published intracellular concentration of 2.3 mM in exponential *E. coli* cells grown with glucose (16). *K*_m_ values for the UDP-GlcNAc and UDP-Glc (both in the range of ∼300 μM) on acnA promoter are also far lower the cellular concentration of these compounds (∼9 and 2.3 mM, respectively) (16), and thus are even more favorable for RNA capping. In addition to NudC, which was shown to uncap NAD-RNAs, there are 13 poorly characterized NUDIX hydrolases in *E. coli*, which can potentially remove capping molecules, hypothetically including FAD and derivatives of UDP (7).

There might be an unexpected connection between capping (and thus potentially gene expression) and the effect of various antibiotics. For example, inhibition of protein synthesis by chloramphenicol or tetracycline, as well as inhibition of cell wall biosynthesis by fosfomycin lead to significant increase of the UDP-GlcNAc concentration (18), potentially changing capping efficiency for transcripts produced from +1U promoters. All tested Rifampicin resistant mutants of RNAP have impaired NAD+ capping activity most likely due to an altered geometry of Rif-pocket. Therefore the reduced fitness of Rifampicin resistant mutants (19), in part, may be attributed to the altered NADylation of RNA resulting in aberrant regulation of RNA stability and thus gene expression. Furthermore, subinhibitory concentrations of Rifampicin may have different effects on production of RNA initiated with NTP and a co-factor, as can be seen from Figure 4A.

Overall our work provides mechanistic insight into the process of prokaryotic capping of RNA by RNAP with the variety of cellular cofactors *in vitro*.
